# Pediatric Telebehavioral Health: A Transformational Shift in Care Delivery in the Era of COVID-19

**DOI:** 10.2196/20157

**Published:** 2020-09-18

**Authors:** Ujjwal Ramtekkar, Jeffrey A Bridge, Glenn Thomas, Eric Butter, Jennifer Reese, Erica Logan, Simon Lin, David Axelson

**Affiliations:** 1 Department of Psychiatry Nationwide Children's Hospital The Ohio State University College of Medicine Columbus, OH United States; 2 Center for Suicide Prevention and Research Departments of Pediatrics, Psychiatry & Behavioral Health The Abigail Wexner Research Institute at Nationwide Children's Hospital Columbus, OH United States; 3 The Abigail Wexner Research Institute at Nationwide Children's Hospital Columbus, OH United States

**Keywords:** telepsychiatry, telebehavioral health, child and adolescent psychiatry, COVID-19

## Abstract

The use of telebehavioral health has been expanding in the past decade to improve access to psychiatric care and address critical shortages in the psychiatric workforce. The coronavirus (COVID-19) pandemic forced a sudden shift from traditional in-person visits to alternative modalities. There are key factors associated with successful transitional and large-scale implementation of telehealth with existing resources. We describe the experience of a large health care system using telehealth technology, and we identify strategies and discuss considerations for long-term sustainability after the pandemic.

## Introduction

As of May 5, 2020, there were approximately 1,012,000 confirmed cases and more than 60,000 deaths in the United States associated with the coronavirus disease (COVID-19) pandemic [1]. COVID-19 profoundly changed behavioral health care delivery. In a period of only weeks, most states issued social distancing guidelines and stay-at-home orders to reduce the spread of the disease. The practice of in-person psychiatric and therapy visits was effectively halted, raising concerns that treatment would be unavailable or clinically compromised. Given the crisis of youth suicide rates, which increased by 69% from 2008 to 2018, and given that hospitalization for all pediatric mental health conditions has increased by nearly 50% during the past decade, the public health implications of lack of care or inadequate treatment could be substantial [2,3]. In this clinical perspective, we discuss how the COVID-19 crisis has exposed opportunities to improve access and care via telehealth technology for youth living with behavioral health conditions. We describe our hospital system’s experience of rapid transition to telebehavioral health services to reduce interruptions in behavioral health care. Finally, we offer suggestions to ensure that telebehavioral health continues to be a priority after the COVID-19 pandemic has passed.

## How a Pandemic Revealed the Value of Telebehavioral Health

An accumulating body of evidence indicates that telebehavioral health is an effective service delivery model for children and adolescents. There are well-established data on the feasibility and acceptability of delivering telebehavioral health services across a variety of clinical settings, and the evidence base for the reliability and clinical outcomes of these services is evolving [4]. Although telebehavioral health is acknowledged as an important tool to reach the broadest population of youth and families, its adoption is not widespread. Barriers to adoption include significant variability in regulations among individual states and regulatory boards; prohibitive federal regulations such as the Ryan Haight act, which restrict prescribing; and lack of reimbursement for certain clinical services (eg, group therapy), provider-to-provider consultation models, and prevention activities; poor distribution of broadband connectivity and availability, which creates a digital divide between urban, rural, and underserved communities; and social determinants, including poverty, literacy, and socioeconomic disadvantage, which affect affordability of telebehavioral health for the vulnerable population that needs but cannot access these services.

As health care organizations, we must be cognizant of the inherent uncertainties in the health care environment, such as sudden regulatory and payor policy changes, shortages of drugs/vaccines due to market disruption, and evolving trends of technology adoption, to be prepared for such contingencies. However, the urgency created by this pandemic forced us to rapidly transition to teletechnology to maintain continuity of care while reducing risk of contagion to both our patients and our workforce. The process of technology evaluation, budget allocation, implementation planning, and operational infrastructure generally lasts several months or years. Our experience demonstrated that the introduction of an effective telehealth program can be greatly accelerated using existing resources. There are a few key factors in the success of this rapid transition:

- Relatively inexpensive, cloud-based video technology solutions are available that do not require additional infrastructure, hardware, or separate end user devices.
- The pediatric population of “digital natives” is highly immersed in digital technology and adept at integrating interactive audioconferencing and videoconferencing tools for socializing with peers, accessing education, and using social media [5]. Using similar modalities to access their health care providers can be a natural extension of the social interaction of younger patients and can even enhance their sense of control and psychological safety.
- On the provider end, significant adjustments are needed to ensure the telebehavioral health experience is natural and authentic while still enabling the collection of sufficient information for clinical decision making. While it is optimal to provide appropriate training to enhance the “webside manner” of our providers, telehealth can be considered as a new venue and a new modality for delivering care, therefore obviating the need for extensive clinical retooling [6].
- Telehealth has the potential to address access issues arising due to geographic distance and critical shortages of pediatric behavioral health specialists [7]. However, simply offering these services is not sufficient; we need to consider social determinants such as barriers to internet connectivity, trust or perception issues based on prior interactions with social services, language barriers, and system capacity to offer virtual interpreter services. These issues should be identified and addressed during implementation of telehealth services to provide equitable access.

## What We Have Learned To Date From Our Experience

As one of the largest pediatric behavioral health departments offering a full continuum of services and serving a large geographic area of the state and parts of neighboring states, it was crucial that we be decisive and nimble in transitioning to telebehavioral health in the context of COVID-19. Our department did have some prior telebehavioral health experience; however, it was limited to approximately 300 annual visits and restricted to medication management services. Within the short span of 3 weeks after completion of implementation, we conducted over 12,500 televisits across a range of services, including psychiatry, psychology, therapy, and autism services. These televisits represent approximately 70% of usual outpatient visits; they included approximately 9500 video visits and 3500 phone and other technology visits, with a trend towards increasing video visits over time. To avoid exposure risk, we decided to transition all ambulatory care from in-person to telehealth. We prioritized children with acute and ongoing needs for continued services and new assessments during this time, while stable cases were initially deferred. For instance, psychiatry services provided medication refills and scheduled later appointments. Outpatient therapy services were initiated for the more acute patients on the waiting list and sustained for established patients. Diagnostic, psychological, and developmental assessment services were initiated for children with greater acuity or risk. Decisions regarding patient priority were based on clinician judgement and family input about the patient’s acuity and comfort with the decision.

**Table 1:** The telebehavioral health transition during the first 4 weeks of implementation.

Week	Unique providers^a^, n	Total telebehavioral health visits, n	Telephone visits, n (%)	Video visits, n (%)
March 22, 2020	314	1735	389 (22.4)	1346 (77.6)
March 29, 2020	407	3102	1180 (38.0)	1922 (62.0)
April 5, 2020	456	3608	981 (27.2)	2627 (72.8)
April 12, 2020	478	4052	828 (20.4)	3224 (79.6)

^a^Unique providers include psychiatrists, psychologists, and masters-level clinicians across the service line.

We adopted a version of the Zoom video platform (Zoom Video Communications) that was compliant with the Health Insurance Portability and Accountability Act (HIPAA) and integrated it into our electronic health record system. All providers were encouraged to use this secure internal solution rather than other, less secure options that were temporarily allowed by the Department of Health and Human Services during the pandemic [8]. Furthermore, we used a coordinated communication strategy to disseminate internally developed training materials and policy updates and provided ongoing live technical support from a virtual command center staffed by internal telehealth content experts within the department.

As expected, caregiver and provider experiences varied depending on previous experience with technology, clinical complexity, and the degree to which clinical interventions needed to be adapted for telebehavioral health encounters. Difficulty reliably connecting to the technology, largely due to rapid implementation, sometimes necessitated the use of alternative modes such as telephone calls to provide service successfully. We also explored multiple technology solutions as backups to the current preferred technology. Due to the minimal planning time, all clinical and operations leaders dedicated resources and applied continuous process improvement based on daily reports. The closure of schools added another layer of challenges for school-based prevention and therapy services requiring coordination with school personnel. While we were able to create workflows to accommodate team-based evaluations, certain components, such as psychological testing, physical exams, or reliable vital signs, could not be accommodated. We also noted a preference for telephone instead of video visits in a subset of families with lower socioeconomic status and in families with significant financial concerns and additional stressors related to COVID-19. Engaging families with few basic resources to effectively participate in care remains challenging. However, the rapid full-scale transition served as a venue to provide behavioral health services on a similar scale without major interruptions in the continuity of clinical care. We recognize the need for careful evaluation of the impact and outcomes of telebehavioral health using the measures outlined by the American Telemedicine Association [9].

## Challenges and Opportunities

The current experience provides significant insights into the feasibility of rapidly scaling and using telebehavioral health services to address the long-standing challenge of access to care and effective use of a scarce specialty workforce. Further innovation is required to operationalize all elements of service delivery that traditionally necessitate in-person interactions to develop a patient-centered hybrid model of in-person and telebehavioral health visits. Conducting a telebehavioral health visit has implications across all stages of clinical operation, ranging from checking in, the consent process, and payment of services to follow-up and remote management of emergencies; all of this necessitates a paradigm shift in which telebehavioral health is regarded as a distinct venue for providing care. In that context, it is also clear that in addition to content expertise, distinct competencies are required for TBH leaders and champions to effectively coordinate transdisciplinary care. The feasibility of ambulatory care experiences must be translated into a variety of activities, such as acute crisis services, group therapy, expanded psychological assessments, and parent-mediated treatments for children with autism.

Furthermore, evolving technologies such as mobile health (apps), artificial intelligence, and immersive virtual reality, which are all currently being developed in isolation, could become an extension of telebehavioral health in conceptualizing “virtual health” as the next step to promote this innovation.

## Conclusions

The COVID-19 pandemic has revealed the inherent value of pediatric telebehavioral health to reach a broad population of young people and families and to meet the clinical needs of people who cannot access in-person behavioral health care due to geography, stigma, or social determinants. Our most recent projections indicate that we will perform over 50,000 telebehavioral health visits within a 6-week period, indicating acceptance of this modality on a large scale. Further analysis of differential adoption as well as the barriers and factors related to social determinants of health is underway. This successful rapid implementation and acceptance on a large scale indicates that telebehavioral health is finding a permanent place as a feasible and efficient way to provide care and partially address the critical shortage of behavioral health care workers. We urge policy makers, health care administrators, clinicians, and other key stakeholders to consider the long-term benefits of investment in this important tool not only as a solution during times of crisis but as a routine component of health care service delivery.

## Abbreviations

COVID-19: coronavirus disease
HIPAA: Health Insurance Portability and Accountability Act
